# Radiomics model based on dual-energy CT venous phase parameters to predict Ki-67 levels in gastrointestinal stromal tumors

**DOI:** 10.3389/fonc.2025.1502062

**Published:** 2025-04-29

**Authors:** Wen-hua Liu, Min Li, Guo-qiang Ren, Zhi-yang Tang, Xiu-hong Shan, Ben-qiang Yang

**Affiliations:** ^1^ Dalian Medical University, Dalian, Liaoning, China; ^2^ Department of Radiology, Jiangsu University affiliated People’s Hospital (Zhenjiang First People’s Hospital), Zhenjiang, Jiangsu, China; ^3^ Department of Radiology, General Hospital of Northern Theater Command, Shenyang, Liaoning, China

**Keywords:** radiomics, tomography, x-ray computed, dual-energy computed tomography, gastrointestinal stromal tumors, iodine density map, atomic number map

## Abstract

**Objective:**

To develop and validate a radiomics model based on the features of the Dual-Energy CT (DECT) venous phase iodine density maps and effective atomic number maps to predict Ki-67 expression levels in gastrointestinal stromal tumors (GISTs).

**Methods:**

A total of 91 patients with GIST were retrospectively analyzed, including 69 patients with low Ki-67 expression (≤5%) and 22 patients with high Ki-67 expression (>5%). Four clinical features (gender, age, maximum tumor diameter, and tumor location) were extracted to construct a clinical model. The venous phase enhanced CT iodine density maps and effective atomic number maps of DSCT were used to build radiomics models. Logistic regression was used to combine radiomics features with clinical features to build a combined model. Finally, the optimal model’s discrimination, calibration, and clinical decision curve were validated using the Bootstrap method.

**Results:**

The combined model was identified as the best model, with high predictive performance. The model’s discrimination had an AUC of 0.982 (95% CI, 0.9603-1). The calibration test showed a Hosmer-Lemeshow test P-value of 0.99. The clinical decision curve demonstrated a probability threshold range of 15% to 98%, with a high net benefit.

**Conclusion:**

The nomogram model combining clinical features and radiomics (iodine density map radscore + effective atomic number map radscore) has the highest accuracy for preoperative prediction of Ki-67 expression in GISTs.

## Introduction

Gastrointestinal stromal tumors (GISTs) are rare mesenchymal tumors originating from the interstitial cells of Cajal (1, 2). GISTs can occur anywhere in the digestive tract and exhibit histological heterogeneity and biological diversity (3), making it challenging to predict their malignant potential. Ki-67, a marker for cellular proliferation, is closely associated with the malignant potential and prognosis of GISTs (4). Conventional methods for detecting Ki-67 rely on surgical or endoscopic fine-needle biopsy samples, which are limited by sampling constraints and operator subjectivity, leading to inaccurate assessments (5). Therefore, developing an reliable, non-invasive method for preoperative Ki-67 evaluation is crucial for optimizing individualized treatment strategies.

Dual-Energy CT (DECT), an emerging CT imaging technology (6, 7), utilizes different energy spectra to differentiate tissues based on their specific attenuation characteristics, offering more detailed tissue information than conventional CT. Multi-parameter DSCT methods—including virtual monochromatic images (VMIs), iodine density maps, electron density maps, and effective atomic number maps—are playing an increasingly important role in tumor evaluation (8, 9). Radiomics allows the extraction of numerous quantitative imaging features, and integrating DECT-based parameters with radiomics can further enhance predictive performance.

## Materials and methods

### Patients

**Figure 1:**
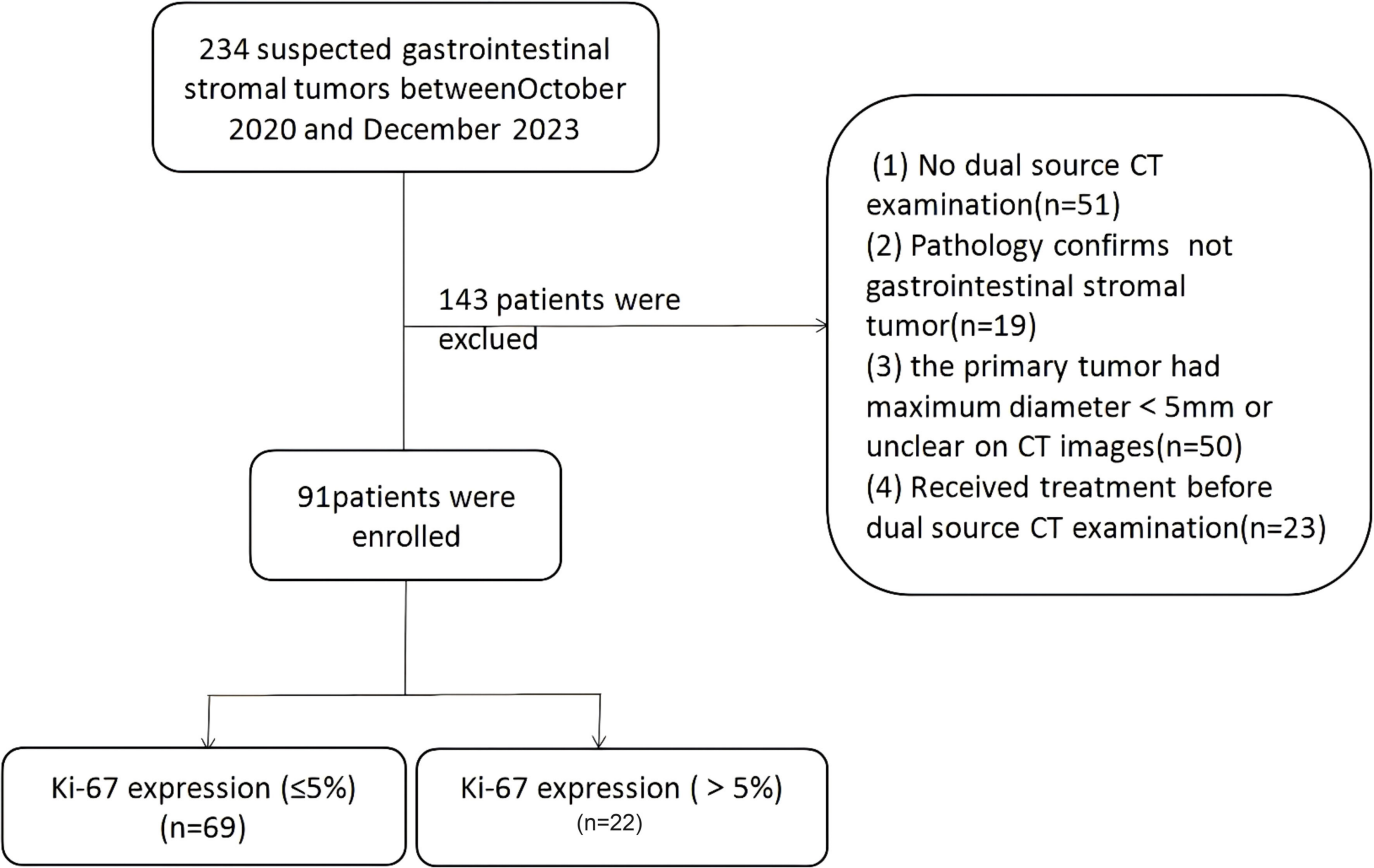
Flowchart of patient inclusion and exclusion in this retrospective study.

**Figure 2:**
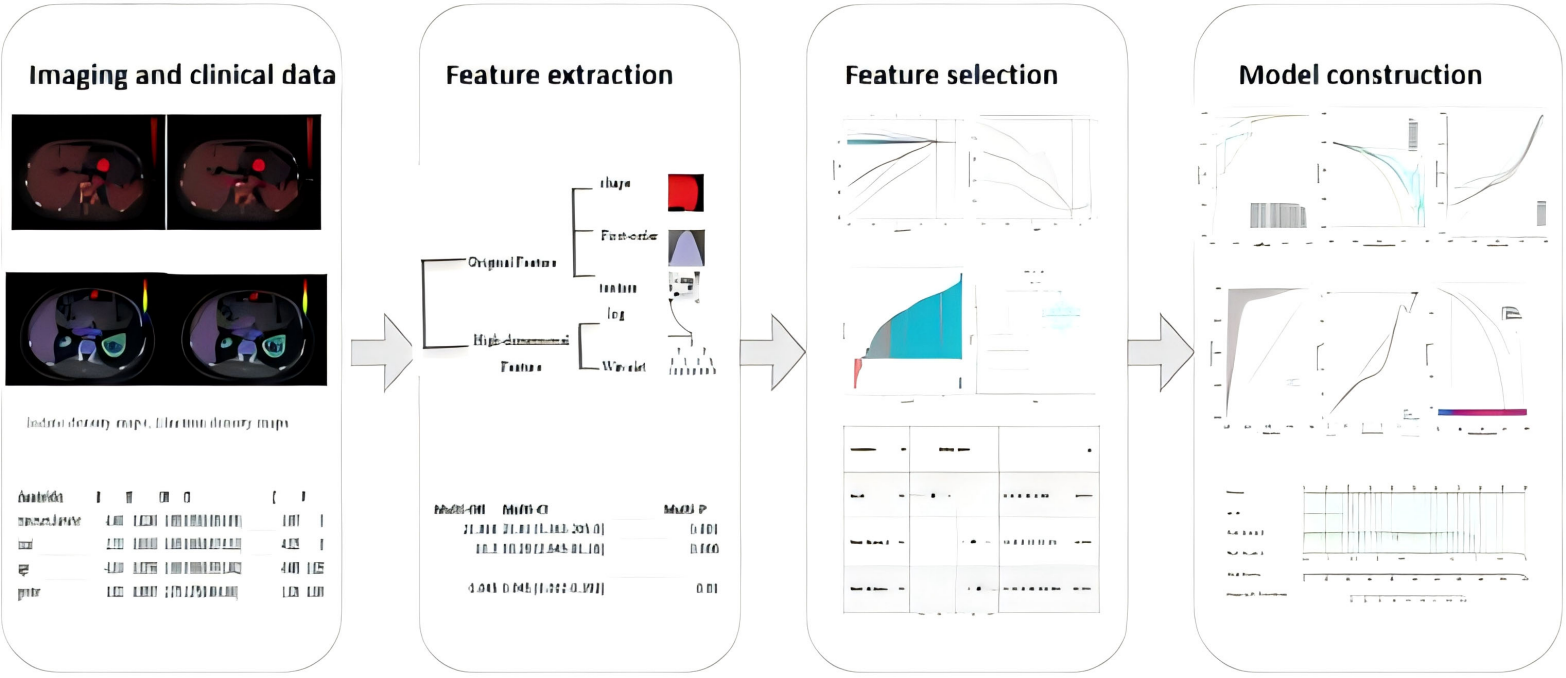
Diagram shows workflow for radiomics and clinical feature.

This retrospective study was approved by the institutional review board, with a waiver of informed consent. All methods were carried out in accordance with policies of the Nature Portfolio journals.This retrospective study was conducted as the workflow indicated (**Figures 1**, **2**). Our study was approved by the institutional review board, and the informed consent was waived. Data were collected from patients diagnosed with GISTs through immunohistochemistry in hospital between October 2020 and December 2023. The inclusion criteria were as follows: (1) patients who have undergone CT scans and are suspected of having gastrointestinal stromal tumors; (2) patients with complete and comprehensive data and pathologically confirmed GISTs, including Ki-67 index. The exclusion criteria include: (1) patients without dual-energy CT examination; (2)without confirmation of GISTs pathologically; (3) patients who received treatment before CT examination; (4) lesions with a diameter less than 5 mm.

### DECT image acquisition

DECT imaging was performed using a third-generation SOMATOM Force CT scanner (Siemens Healthineers, Forchheim, Germany). The scanning parameters were as follows: Tube A and Tube B voltages were set at 100 kVp and Sn150 kVp, respectively, with tube currents of 80 and 41 mAs. Automatic current modulation was activated. A non-ionic contrast agent, iopamidol(Jiangsu Hengrui Company,China), was injected. Arterial phase image acquisition was initiated when the CT value of the abdominal aorta at the level of the diaphragm reached 100 HU. Venous phase images were acquired in dual-energy mode 35 seconds after the arterial phase.

### CT image segmentation

We processed the dual-energy images on a Siemens workstation (Syngo.via VB20) to obtain iodine maps and effective atomic number maps. The venous phase CT images of all included samples were exported in DICOM format and imported into ITK-Snap software (an open-source software, www.itk-snap.org) for automatic delineation of the regions of interest (ROI). After delineation, the ROIs were corrected by an abdominal radiologist with 10 years of experience.

### Image normalization and feature extraction

The images were normalized before feature extraction. Radiomics features based on Dual-Energy CT (DECT) were extracted using the Pyradiomics package (version 2.2.0) in Python (version 3.7) following to the guidelines of the Image Biomarker Standardization Initiative (IBSI) (10). The reproducibility of radiomics features was tested using a random sample of 30 CT images, comparing automatic and manual delineation. The intraclass correlation coefficient (ICC) was used to evaluate the consistency of ROI delineation between the two methods. Good consistency was defined as ICC > 0.75, and features with ICC < 0.75 were excluded.

### Feature extraction

Radiomics features were extracted using the open-source Python package Pyradiomics (version 2.2.0) (https://github.com/Radiomics/pyradiomics), yielding a total of 960 radiomics features from the Volume of Interest (VOI) regions. These features included:14 shape features, 18 first-order statistics features, 68 texture features, 860 high-dimensional features. High-dimensional features comprised: 18 first-order features derived from log-sigma transformations, 154 texture features derived from log-sigma transformations, 18 first-order features derived from wavelet transformations, 670 texture features derived from wavelet transformations.

### Clinical model, radiomics model, and combined model development

Univariate logistic regression analysis was performed to identify significant features (p < 0.05), which were then included in a multivariate logistic regression analysis to develop predictive models. Clinical characteristics (age, gender, tumor location, and maximum tumor diameter) were used to establish the clinical model. From the venous phase enhanced CT iodine density maps and effective atomic number maps of dual-energy CT, 960 radiomics features were extracted and imported into DCPM (V4.01, Jingding Medical Technology Co., Ltd.). Dimensionality reduction was performed using the intraclass correlation coefficient (ICC) and least absolute shrinkage and selection operator (LASSO) algorithm, resulting in radscore values for the iodine density and effective atomic number maps, which were used to develop the radiomics model. Logistic regression was then used to combine radiomics features with clinical features to build the combined model. The performance of each model was evaluated using the area under the receiver operating characteristic curve (AUC). The calibration of each model was assessed using the Hosmer-Lemeshow test, and the differences in AUC values between models were compared using the DeLong test to identify the optimal model. Finally, the discrimination, calibration, and clinical utility of the optimal model were validated using the Bootstrap method.

### Statistical analysis

Data analysis was performed using the R software package (version 4.2.1) and DCPM (V4.01, Jingding Medical Technology Co., Ltd.). Normality of continuous data was tested using the Kolmogorov-Smirnov (K-S) test and the Shapiro-Wilk (S-W) test. Normally distributed data were analyzed using a two-sided independent samples t-test. Non-normally distributed data were compared using the Mann-Whitney U test. Categorical variables were analyzed using Fisher’s exact test. Features with statistical significance in univariate analysis were further subjected to binary logistic multivariate regression analysis, with p < 0.05 considered statistically significant. Model performance was evaluated using the area under the receiver operating characteristic curve (AUC), and calibration was assessed using the Hosmer-Lemeshow test. The DeLong test was used to compare differences in AUC values between models.

## Results

### Comparison between Ki-67 high and low expression groups

Ultimately,91 patients (47 females and 44 males) with an average age of 62.15 ± 8.86 years were selected for retrospective analysis (**Table 1**). Among them, 74 GISTs were located in the stomach and 17 were located in other parts of the gastrointestinal tract.Patients were divided high Ki-67 expression and low Ki-67 expression groups, and clinical data were compared based on the expression levels of Ki-67 Comparisons of clinical and radiomics features between the two groups (**Table 2**). Comparisons of clinical and radiomics features between the two groups revealed that tumor location differed significantly in both univariate and multivariate logistic regression analyses. Tumor size showed statistical significance in the univariate analysis (p < 0.05) but was not significant in the multivariate analysis. Age and gender did not show any statistically significant differences in either univariate or multivariate analyses.

**Table 1:** Clinical characteristics of patients.

	[ALL] N=91	N=22 Ki-67>5%	N=69 Ki-67 ≤5%	Pvalue
Gender:				0.161
male	44 (48.35%)	14 (63.64%)	30 (43.48%)	
female	47 (51.65%)	8 (36.36%)	39 (56.52%)	
Age	62.15 (8.86)	62.95 (10.30)	61.90 (8.41)	0.665
Local:				<0.001
stomach	74 (81.32%)	10 (45.45%)	64 (92.75%)	
Outside the stomach	17 (18.68%)	12 (54.55%)	5 (7.25%)	
maximum.diameter	24.00 [16.00;38.00]	41.00 [26.25;75.25]	21.00 [14.00;31.00]	<0.001

**Table 2:** All Characteristics of Patients Single factor logistic regression and multiple logistic regression.

	Characteristics	Uning-OR	Uni-CI	Uni-P	Multi-OR	Multi-CI	Multi-P
1	Rad_Score.I.	10.42	10.42 (4.212-34.43)	0	21.816	21.81 (5.363-205.0)	0.001
2	Rad_Score.Z.	9.834	9.834 (3.866-33.31)	0	10.3	10.29 (2.645-81.16)	0.006
3	maximum.diameter	0.955	0.955 (0.929-0.976)	0			
4	local	0.065	0.065 (0.017-0.213)	0	0.045	0.045 (0.002-0.392)	0.01
5	age	0.986	0.986 (0.933-1.042)	0.625			
6	gender	2.275	2.275 (0.86-6.366)	0.104			

I:iodine density maps Z:effective atomic number maps.

### Feature selection and model development

**Figure 3:**
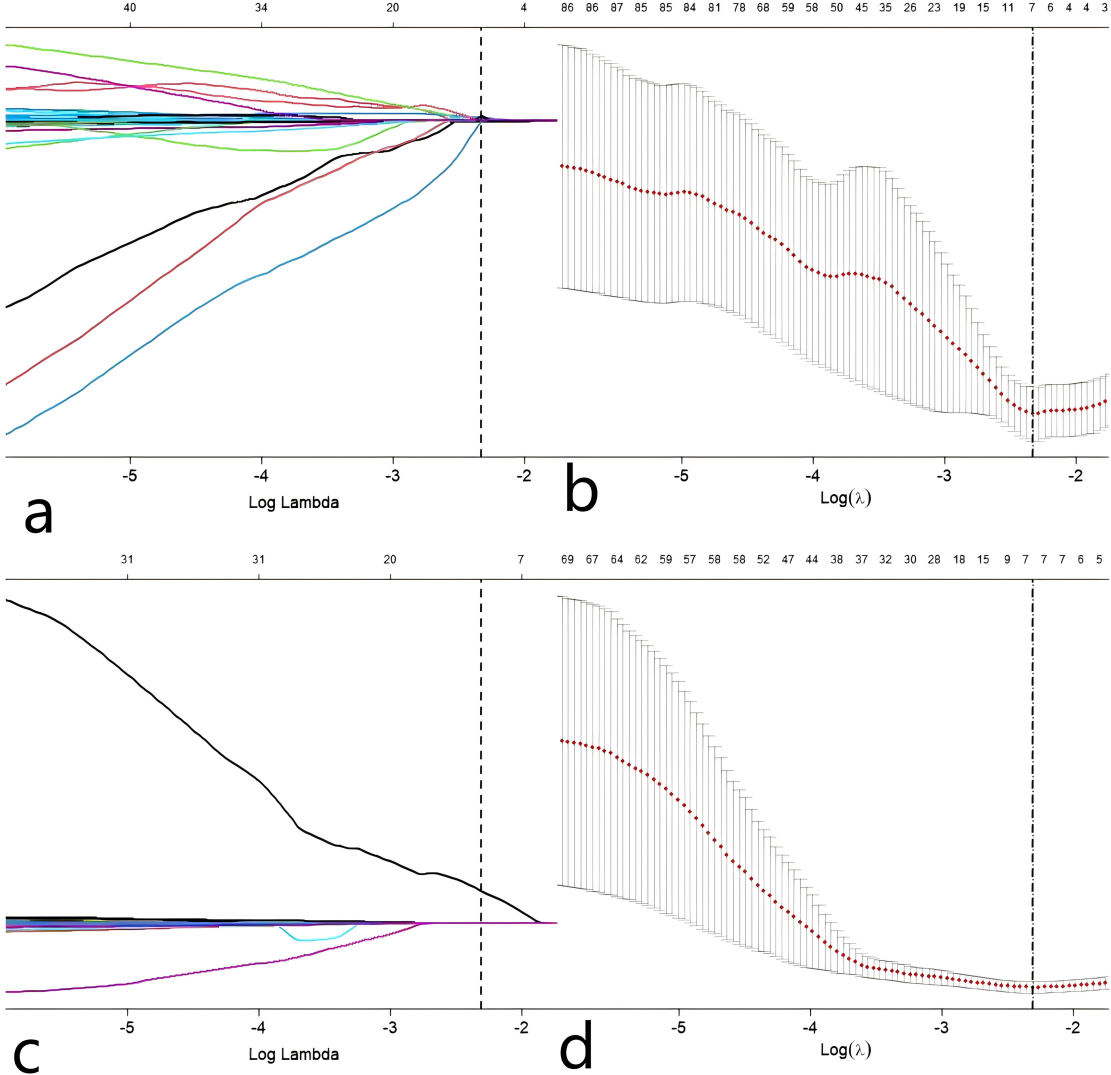
Predictor selection(Z **a**, **b**) using the LASSO regression analysis with tenfold cross-validation. parameter (lambda) selection of deviance in the LASSO regression based on the minimum criteria (left dotted line) and the 1-SE criteria (right dotted line). In the present study, predictor’s selection was according to the 1-SE. Predictor selection(I **c**, **d**) using the LASSO regression analysis with tenfold cross-validation. parameter (lambda) selection of deviance in the LASSO regression based on the minimum criteria (left dotted line) and the 1-SE criteria (right dotted line). In the present study, predictor’s selection was according to the 1-SE.

**Figure 4:**
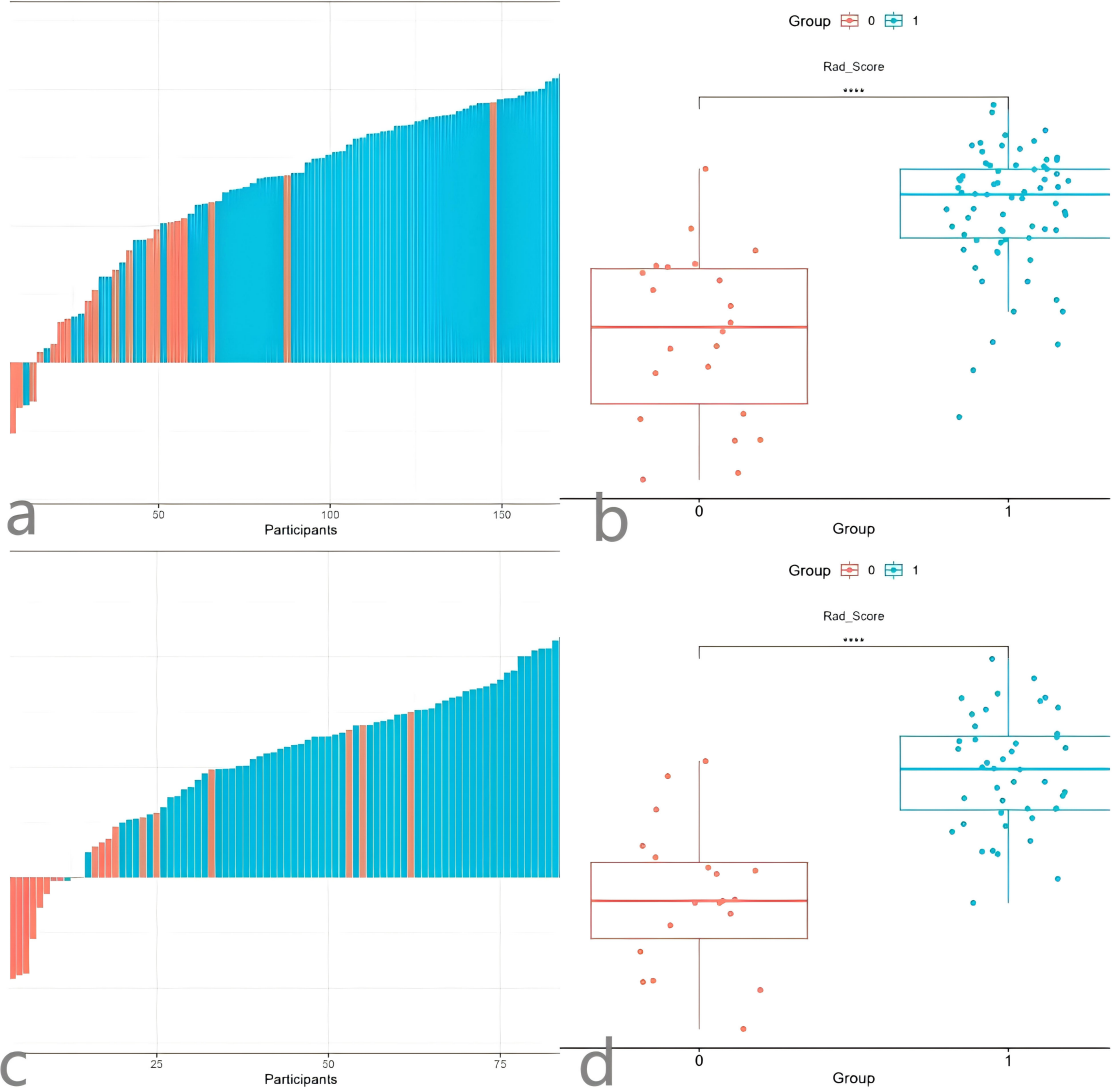
Waterfall Plot **(a)** and comparison Plot **(b)** of the radiomics Z model. Waterfall Plot **(c)** and comparison Plot **(d)** of the radiomicsI model.

We also tested the consistency of 670 radiomics features from each of the iodine density maps and effective atomic number maps. Initially, the radiomics features extracted from each phase were subjected to feature selection and dimensionality reduction using the LASSO algorithm (**Figure 3**). Based on the selection of predictors by log(lambda) and the 1-SE criteria (**Figure 3**), five radiomics features from the iodine density map and 2 from the effective atomic number map were ultimately selected. These selected radiomics features were used to calculate two radscores. Multivariate logistic regression was then used to establish radiomics models for each phase. The radscores effectively distinguished between high and low Ki-67 expression cases (**Figure 4**). The AUC values were used to compare the models within each phase to identify the optimal model. Finally, six prediction models were established, including one clinical model, two radiomics models (iodine density map and effective atomic number map), and three combined models (clinical + iodine density map; clinical + effective atomic number map; clinical + iodine density map + effective atomic number map). The results showed that there was good consistency (ICCs > 0.75) of extracted tumor features.

### Model comparison and validation

**Figure 5:**
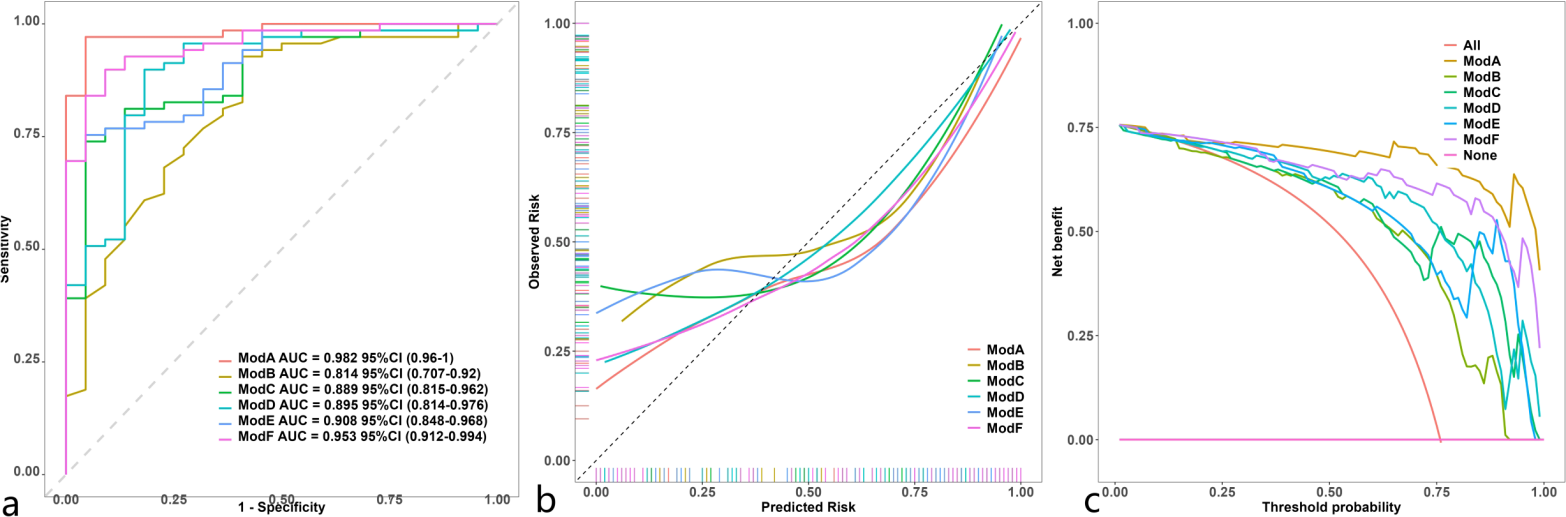
Comparison of ROC **(a)** in 6 model ModelA: Clinical+Rad_Score.Z.+Rad_Score.I.ModelB: Clinical.IModelC: Rad_Score.Z. ModelD: Rad_Score.I. ModelE: Clinical+Rad_Score.Z.ModelF: Clinical+Rad_Score.I.). Comparison of calibration curve **(b)** in 6 model (ModelA: Clinical+Rad_Score.Z.+Rad_Score.I.ModelB: Clinical.IModelC: Rad_Score.Z. ModelD: Rad_Score.I. ModelE: Clinical+Rad_Score.Z.ModelF: Clinical+Rad_Score.I.). Comparison of decision curve analysis **(c)** in 6 model (ModelA: Clinical+Rad_Score.Z.+Rad_Score. I.ModelB: Clinical.IModelC: Rad_Score.Z. ModelD: Rad_Score.I. ModelE: Clinical+Rad_Score.Z.ModelF: Clinical+Rad_Score.I.).

**Figure 6:**
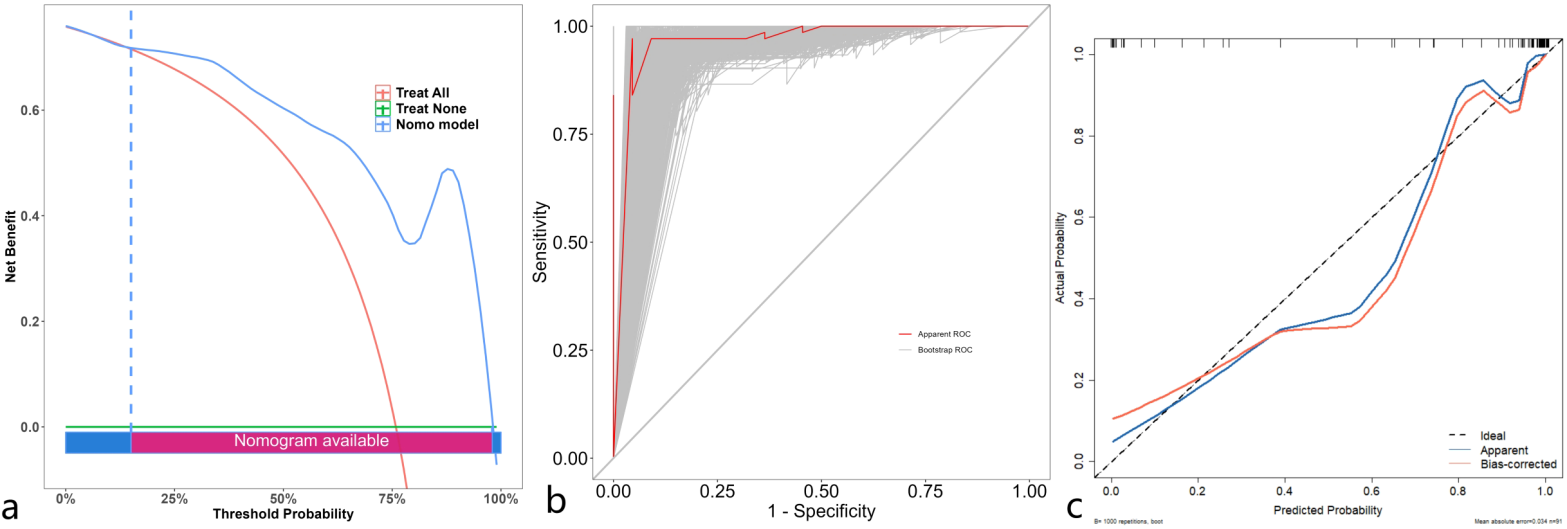
Decision curve **(a)** analysis of the optimal model validated through 1000 bootstrp (threshold range of 15% to 98%). ROC **(b)** of the optimal model validated through 1000 bootstrp. Calibration curve **(c)** of the optimal model validated through 1000 bootstrp.

The discrimination, calibration, and clinical applicability of the six models are shown (**Figure 5**). The ROC curves were compared using the DeLong test (**Table 3**), revealing statistical differences between the combined clinical + iodine density map + effective atomic number map model and the other four models, but no statistical difference between the clinical + iodine density map model and the clinical model. The clinical + iodine density map + effective atomic number map model was identified as the best model. The optimal model’s discrimination, calibration, and clinical decision curve were validated using the Bootstrap (1000 times) method. This model had the highest predictive performance with an AUC of 0.982 (95% CI, 0.9603-1) (**Figure 6**). The Hosmer-Lemeshow test showed a calibration P-value of 0.99 (**Figure 6**). The clinical decision curve indicated a probability threshold range of 15% to 98%, with high net benefit (**Figure 6**). The optimal model was visualized using a nomogram that combined clinical features, iodine density map, and effective atomic number map radiomics.

**Table 3:** The ROC curves of 6 model compared using the DeLong test.

1	Model	Model	Pvalue
2	Clinical+Rad_Score.Z.+Rad_Score.I.	Clinical	0
3	Clinical+Rad_Score.Z.+Rad_Score.I.	Rad_Score.Z.	0
4	Clinical+Rad_Score.Z.+Rad_Score.I.	Rad_Score.I.	0.03
5	Clinical+Rad_Score.Z.+Rad_Score.I.	Clinical+Rad_Score.Z.	0.01
6	Clinical+Rad_Score.Z.+Rad_Score.I.	Clinical+Rad_Score.I.	0.06

## Discussion

Dual-energy CT iodine density maps and effective atomic number maps with radiomic and clinical features for predicting preoperative Ki-67 expression in gastrointestinal stromal tumors (GISTs). Our findings demonstrate that models incorporating both clinical and imaging radiomic features achieve high predictive accuracy, with the highest AUC reaching 0.982. Notably There was no significant difference in accuracy between the best models using clinical + iodine density map radscore and clinical + iodine density map radscore + effective atomic number radscore. These findings suggest that models utilizing dual-energy CT iodine density and effective atomic number maps hold significant clinical value for non-invasive preoperative assessment of Ki-67 expression in GISTs, thereby avoiding the risks associated with biopsy-induced tumor rupture and dissemination. We developed and validated the best-performing combined model using bootstrap resampling (1,000 iterations), with a calibration plot demonstrating excellent agreement (AUC = 0.982, 95% CI: 0.9603–1). Clinical decision curve analysis (DCA) indicated a probability threshold range of 15%–98%, yielding a high net benefit, making it a reliable tool for preoperative Ki-67 assessment and clinical decision-making.

This study represents the first preoperative prediction model for Ki-67 expression in GISTs using dual-energy CT iodine density and effective atomic number maps with high accuracy. Previous studies primarily relied on traditional CT features to assess Ki-67 expression in GISTs. However, DECT has emerged as a powerful non-invasive functional imaging modality, providing objective and quantitative information for disease diagnosis and differentiation (11, 12). Unlike conventional imaging features, DECT enables the acquisition of iodine density maps, electron density maps, and effective atomic number maps. Iodine maps utilize dual-energy X-ray imaging to differentiate substances based on their attenuation characteristics at different energies, while electron density and effective atomic number maps generate atomic number distribution maps of substances, offering detailed information on tissue composition. Previous research has demonstrated correlations between DECT iodine density, electron density, effective atomic number parameters, and Ki-67 expression in cervical cancer, gastric adenocarcinoma, and breast cancer (13–15). Our study confirmed that models incorporating iodine density and effective atomic number maps are effective in predicting Ki-67 levels in GISTs.

GISTs occur in various locations and tissue types, leading to differences in prognosis and response to treatment. Current risk stratification measures, such as the modified American Gastrointestinal Stromal Tumor Risk Classification Criteria, fail to comprehensively assess tumor behavior, as low-risk GISTs can still metastasize or recur. Ki-67 is a well-established marker of tumor proliferation. A meta-analysis (16) has shown that higher Ki-67 indices are associated with poorer postoperative outcomes and higher recurrence risks in GISTs.The selection criteria for Ki-67 thresholds in GISTs vary in previous studies, ranging from 5% to 10% (17–20); with 5% being commonly used. Our study adopted this threshold and developed predictive models, and showed favorable outcomes. Traditional Ki-67 detection methods rely on surgical or fine-needle biopsy samples, which may suffer from sampling heterogeneity and limited accuracy. Our radiomics-based approach offers a non-invasive alternative for preoperative Ki-67 evaluation, which could be instrumental in guiding treatment decisions.

Therefore, developing an accurate non-invasive tool for preoperative assessment of Ki-67 expression in GIST patients is crucial. Our findings align with previous studies indicating higher Ki-67 expression levels in extra gastric GISTs compared to gastric GISTs (21), and we utilized this information to build predictive models. Tumor size has also been identified as an important predictor of Ki-67 expression; larger tumors tend to have higher Ki-67 expression probabilities. However, in our multivariate logistic regression analysis, tumor size was excluded, possibly due to sample size limitations. Radiomics has been widely used to integrate diverse tumor phenotypes for developing predictive models in tumor diagnosis, grading, treatment response assessment, and prognosis prediction (22–24). In the existing body of literature, investigations pertaining to the employment of radiomics for the prediction of Ki - 67 expression levels within gastrointestinal stromal tumors (GISTs) have predominantly been predicated on the imaging manifestations of conventional computed tomography (CT). Conversely, the present study is uniquely founded upon the iodine - based maps and atomic number maps that are generated subsequent to the post - processing of dual - source CT data. Notably, the Area Under the Curve (AUC) of the optimal prediction model derived from our research substantially exceeds the corresponding outcomes reported in the previously surveyed literature (17–20, 22).

However, our study has several limitations: retrospective design and single-center data, which may introduce selection bias. Only venous phase images were analyzed, potentially limiting the model’s generalizability. Therefore, future studies should explore multicenter and multiphase approaches to validate these findings further.

## Data Availability

The datasets presented in this study can be found in online repositories. The names of the repository/repositories and accession number(s) can be found in the article/supplementary material.
